# 2,2′-(1,10-Phenanthrolin-2-ylimino)di­ethanol

**DOI:** 10.1107/S1600536809004929

**Published:** 2009-02-18

**Authors:** Xia Jin, Jin Min Li

**Affiliations:** a Chemistry and Chemical Engineering College, Shanxi Datong University, Datong 037009, People’s Republic of China

## Abstract

In the title compound, C_16_H_17_N_3_O_2_, symmetry-related mol­ecules are linked into one-dimensional chains along the *a* axis by a combination of inter­molecular O—H⋯N and O—H⋯O hydrogen bonds and weak π–π stacking inter­actions with a centroid–centroid distance of 3.5494 (12) Å.

## Related literature

For recent crystal structure reports on the complexes formed with derivatives of 1,10-phenanthroline, see for example: Li *et al.* (2008 ▶) and Zhang *et al.* (2008 ▶).
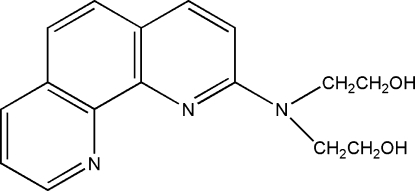


## Experimental

### 

#### Crystal data

C_16_H_17_N_3_O_2_
*M* *_r_* = 283.33
Monoclinic, 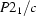

*a* = 4.8480 (10) Å
*b* = 14.854 (3) Å
*c* = 18.858 (4) Å
β = 93.523 (3)°
*V* = 1355.5 (5) Å^3^
*Z* = 4
Mo *K*α radiation
μ = 0.09 mm^−1^
*T* = 298 K
0.54 × 0.32 × 0.20 mm

#### Data collection

Bruker SMART APEX CCD diffractometer
Absorption correction: multi-scan (*SADABS*; Sheldrick, 1996 ▶) *T*_min_ = 0.951, *T*_max_ = 0.982
7520 measured reflections
2765 independent reflections
2149 reflections with *I* > 2σ(*I*)
*R*_int_ = 0.030

#### Refinement

*R*[*F* ^2^ > 2σ(*F* ^2^)] = 0.047
*wR*(*F* ^2^) = 0.132
*S* = 1.03
2765 reflections
190 parameters
H-atom parameters constrained
Δρ_max_ = 0.24 e Å^−3^
Δρ_min_ = −0.16 e Å^−3^

### 

Data collection: *SMART* (Bruker, 1997 ▶); cell refinement: *SAINT* (Bruker, 1997 ▶); data reduction: *SAINT*; program(s) used to solve structure: *SHELXTL* (Sheldrick, 2008 ▶); program(s) used to refine structure: *SHELXTL*; molecular graphics: *SHELXTL*; software used to prepare material for publication: *SHELXTL*.

## Supplementary Material

Crystal structure: contains datablocks I, global. DOI: 10.1107/S1600536809004929/lh2772sup1.cif

Structure factors: contains datablocks I. DOI: 10.1107/S1600536809004929/lh2772Isup2.hkl

Additional supplementary materials:  crystallographic information; 3D view; checkCIF report

## Figures and Tables

**Table 1:** Hydrogen-bond geometry (Å, °)

*D*—H⋯*A*	*D*—H	H⋯*A*	*D*⋯*A*	*D*—H⋯*A*
O2—H5⋯O1^i^	0.91	1.91	2.8254 (17)	177
O1—H4⋯N1^ii^	0.83	2.08	2.8676 (18)	158

Symmetry codes: (i) 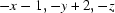; (ii) 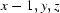.

**Fig. 1.**
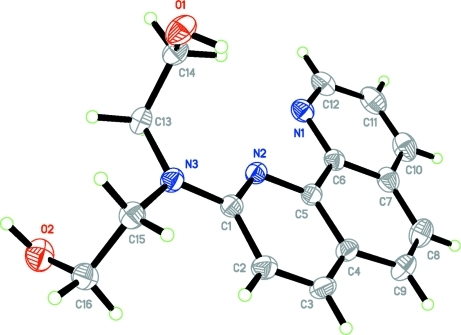
The molecular structure of (I), showing the the atom numbering scheme with thermal ellipsoids drawn at the 30% probability level

**Fig. 2.**
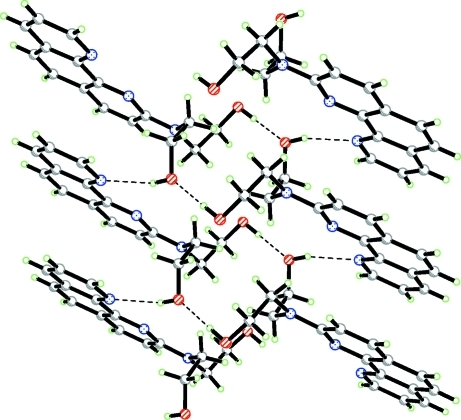
Part of the crystal structure of (I) showing hydrogen bonds (dashed lines) between symmetry realted molecules which form a one-dimensional chain along the a axis.

## supplementary crystallographic information

### Comment

Derivatives of 1,10-phenanthroline play a vital role in a modern coordination chemistry and a number of complexes have been published with these types of compounds as ligands [see for example the recent publications by Li *et al.* (2008) and Zhang *et al.* (2008)]. An interest in designing new derivatives led to the synthesis the title compound, (I), and its crystal structure is reported herein.

The molecular structure of (I) is shown in Fig. 1. In the crystal structure, intermolecular O—H···N and O—H···O hydrogen bonds (see Table 1) connect symmetry related molecules to form 1-D chains along the a axis (see Fig. 2). In addition, within these chain there are weak π-π stacking interactions between symmetry related pyridyl rings, with the relevant distances being *Cg*1···*Cg*2^iii^ = 3.5494 (12) Å, *Cg*1···*Cg*2^iii^_perp_ = 3.464 Å and α = 1.48° [symmetry code (iii) 1+*x*, *y*, *z*; *Cg*1 and *Cg*2 are the centroids of the C6C7C10-C12/N1 and C1—C5/N2 rings, respectively; *Cg*1···*Cg*2^iii^_perp_ is the perpendicular distance from ring *Cg*1 to ring *Cg*2^iii^; α is the dihedral angle between ring plane *Cg*1 and ring plane *Cg*2^iii^].

### Experimental

2-Diethanolamine-1,10-phenanthroline (0.0523 g, 0.185 mmol) was dissolved into 10 ml methanol and the yellow single crystals were obtained after the solution had been allowed to stand at room temperature for one week.

### Refinement

The H atoms of hydroxyl groups were found in a difference Fourier map and were included in 'as found' positions with d(O—H) = 0.83 & 0.91 Å; all other H atoms were placed in calculated positions with C—H = 0.93–0.97 Å. All H atoms were refined as riding, with *U*_iso_(H) = 1.5_eq_(O) for hydroxyl group and *U*_iso_(H) = 1.2*U*_eq_(C) for other groups.

### Crystal data

**Table:**

C_16_H_17_N_3_O_2_	*F*(000) = 600
*M**_r_* = 283.33	*D*_x_ = 1.388 Mg m^−^^3^
Monoclinic, *P*2_1_/*c*	Mo *K*α radiation, λ = 0.71073 Å
Hall symbol: -P 2ybc	Cell parameters from 2381 reflections
*a* = 4.848 (1) Å	θ = 2.6–27.3°
*b* = 14.854 (3) Å	µ = 0.09 mm^−^^1^
*c* = 18.858 (4) Å	*T* = 298 K
β = 93.523 (3)°	Block, yellow
*V* = 1355.5 (5) Å^3^	0.54 × 0.32 × 0.20 mm
*Z* = 4	

### Data collection

**Table:**

Bruker SMART APEX CCD diffractometer	2765 independent reflections
Radiation source: fine-focus sealed tube	2149 reflections with *I* > 2σ(*I*)
graphite	*R*_int_ = 0.030
φ and ω scans	θ_max_ = 26.5°, θ_min_ = 1.8°
Absorption correction: multi-scan (*SADABS*; Sheldrick, 1996)	*h* = −5→6
*T*_min_ = 0.951, *T*_max_ = 0.982	*k* = −12→18
7520 measured reflections	*l* = −23→23

### Refinement

**Table:**

Refinement on *F*^2^	Primary atom site location: structure-invariant direct methods
Least-squares matrix: full	Secondary atom site location: difference Fourier map
*R*[*F*^2^ > 2σ(*F*^2^)] = 0.047	Hydrogen site location: inferred from neighbouring sites
*wR*(*F*^2^) = 0.132	H-atom parameters constrained
*S* = 1.02	*w* = 1/[σ^2^(*F*_o_^2^) + (0.0762*P*)^2^ + 0.108*P*] where *P* = (*F*_o_^2^ + 2*F*_c_^2^)/3
2765 reflections	(Δ/σ)_max_ = 0.008
190 parameters	Δρ_max_ = 0.24 e Å^−^^3^
0 restraints	Δρ_min_ = −0.16 e Å^−^^3^

### Special details

**Table:**

Geometry. All e.s.d.'s (except the e.s.d. in the dihedral angle between two l.s. planes) are estimated using the full covariance matrix. The cell e.s.d.'s are taken into account individually in the estimation of e.s.d.'s in distances, angles and torsion angles; correlations between e.s.d.'s in cell parameters are only used when they are defined by crystal symmetry. An approximate (isotropic) treatment of cell e.s.d.'s is used for estimating e.s.d.'s involving l.s. planes.
Refinement. Refinement of *F*^2^ against ALL reflections. The weighted *R*-factor *wR* and goodness of fit *S* are based on *F*^2^, conventional *R*-factors *R* are based on *F*, with *F* set to zero for negative *F*^2^. The threshold expression of *F*^2^ > σ(*F*^2^) is used only for calculating *R*-factors(gt) *etc*. and is not relevant to the choice of reflections for refinement. *R*-factors based on *F*^2^ are statistically about twice as large as those based on *F*, and *R*- factors based on ALL data will be even larger.

### Fractional atomic coordinates and isotropic or equivalent isotropic displacement parameters (Å^2^)

**Table:**

	*x*	*y*	*z*	*U*_iso_*/*U*_eq_	
C1	−0.1483 (3)	0.89542 (10)	0.18581 (7)	0.0333 (3)	
C2	−0.2322 (3)	0.81999 (11)	0.22608 (8)	0.0408 (4)	
H2	−0.3697	0.7814	0.2077	0.049*	
C3	−0.1090 (4)	0.80542 (11)	0.29126 (8)	0.0439 (4)	
H3	−0.1645	0.7569	0.3181	0.053*	
C4	0.1020 (3)	0.86265 (10)	0.31895 (8)	0.0391 (4)	
C5	0.1770 (3)	0.93444 (10)	0.27517 (7)	0.0336 (3)	
C6	0.3910 (3)	0.99566 (10)	0.30193 (7)	0.0356 (4)	
C7	0.5185 (3)	0.98199 (12)	0.37060 (8)	0.0460 (4)	
C8	0.4363 (4)	0.90765 (14)	0.41232 (9)	0.0597 (5)	
H8	0.5227	0.8980	0.4571	0.072*	
C9	0.2361 (4)	0.85156 (13)	0.38776 (9)	0.0553 (5)	
H9	0.1833	0.8043	0.4163	0.066*	
C10	0.7237 (4)	1.04302 (14)	0.39446 (10)	0.0593 (5)	
H10	0.8122	1.0362	0.4393	0.071*	
C11	0.7935 (4)	1.11242 (14)	0.35196 (11)	0.0598 (5)	
H11	0.9289	1.1536	0.3673	0.072*	
C12	0.6584 (4)	1.12040 (12)	0.28535 (10)	0.0494 (4)	
H12	0.7079	1.1679	0.2567	0.059*	
C13	−0.1736 (3)	0.99054 (11)	0.08028 (7)	0.0387 (4)	
H13A	−0.2437	0.9841	0.0313	0.046*	
H13B	0.0264	0.9875	0.0811	0.046*	
C14	−0.2546 (3)	1.08165 (11)	0.10638 (8)	0.0402 (4)	
H14A	−0.1674	1.0912	0.1535	0.048*	
H14B	−0.1856	1.1273	0.0752	0.048*	
C15	−0.4906 (3)	0.85897 (10)	0.08751 (8)	0.0392 (4)	
H15A	−0.6007	0.8955	0.0538	0.047*	
H15B	−0.6107	0.8389	0.1236	0.047*	
C16	−0.3880 (4)	0.77747 (11)	0.04926 (8)	0.0465 (4)	
H16A	−0.3010	0.7366	0.0840	0.056*	
H16B	−0.5452	0.7465	0.0262	0.056*	
N1	0.4640 (3)	1.06489 (9)	0.25999 (7)	0.0415 (3)	
N2	0.0550 (2)	0.94980 (8)	0.20978 (6)	0.0333 (3)	
N3	−0.2733 (3)	0.91507 (8)	0.12111 (6)	0.0374 (3)	
O1	−0.5458 (2)	1.09241 (8)	0.10935 (6)	0.0499 (3)	
H4	−0.5826	1.0760	0.1499	0.075*	
O2	−0.1971 (3)	0.79774 (9)	−0.00230 (6)	0.0586 (4)	
H5	−0.2838	0.8313	−0.0377	0.088*	

### Atomic displacement parameters (Å^2^)

**Table:**

	*U*^11^	*U*^22^	*U*^33^	*U*^12^	*U*^13^	*U*^23^
C1	0.0325 (8)	0.0333 (8)	0.0345 (7)	0.0036 (6)	0.0044 (6)	−0.0001 (6)
C2	0.0433 (9)	0.0355 (8)	0.0436 (8)	−0.0043 (7)	0.0027 (7)	0.0017 (7)
C3	0.0524 (10)	0.0343 (9)	0.0457 (9)	0.0012 (7)	0.0090 (8)	0.0101 (7)
C4	0.0435 (9)	0.0375 (8)	0.0366 (8)	0.0056 (7)	0.0032 (7)	0.0059 (6)
C5	0.0338 (8)	0.0350 (8)	0.0322 (7)	0.0063 (6)	0.0043 (6)	0.0014 (6)
C6	0.0331 (9)	0.0370 (8)	0.0369 (8)	0.0080 (6)	0.0041 (6)	−0.0032 (6)
C7	0.0436 (10)	0.0529 (10)	0.0407 (8)	0.0079 (8)	−0.0035 (7)	−0.0069 (7)
C8	0.0682 (13)	0.0704 (13)	0.0385 (9)	0.0051 (10)	−0.0131 (8)	0.0081 (8)
C9	0.0676 (13)	0.0566 (11)	0.0411 (9)	0.0044 (9)	−0.0018 (8)	0.0161 (8)
C10	0.0505 (11)	0.0736 (14)	0.0522 (10)	0.0062 (10)	−0.0103 (8)	−0.0156 (9)
C11	0.0466 (11)	0.0627 (12)	0.0694 (12)	−0.0077 (9)	−0.0009 (9)	−0.0228 (10)
C12	0.0458 (10)	0.0443 (10)	0.0589 (10)	−0.0045 (8)	0.0096 (8)	−0.0127 (8)
C13	0.0360 (9)	0.0494 (9)	0.0306 (7)	−0.0033 (7)	0.0005 (6)	0.0062 (6)
C14	0.0358 (9)	0.0421 (9)	0.0423 (8)	−0.0042 (7)	−0.0003 (7)	0.0088 (6)
C15	0.0342 (9)	0.0430 (9)	0.0398 (8)	0.0002 (7)	−0.0022 (6)	0.0012 (6)
C16	0.0522 (11)	0.0417 (9)	0.0448 (9)	0.0025 (7)	−0.0047 (8)	−0.0014 (7)
N1	0.0383 (8)	0.0410 (7)	0.0455 (7)	−0.0010 (6)	0.0062 (6)	−0.0048 (6)
N2	0.0330 (7)	0.0344 (7)	0.0327 (6)	0.0026 (5)	0.0039 (5)	0.0027 (5)
N3	0.0404 (7)	0.0374 (7)	0.0337 (6)	−0.0034 (6)	−0.0025 (5)	0.0031 (5)
O1	0.0394 (7)	0.0602 (8)	0.0502 (7)	0.0085 (5)	0.0040 (5)	0.0163 (5)
O2	0.0552 (8)	0.0697 (9)	0.0514 (7)	0.0186 (6)	0.0069 (6)	−0.0034 (6)

### Geometric parameters (Å, °)

**Table:**

C1—N2	1.3319 (19)	C11—C12	1.386 (3)
C1—N3	1.3603 (18)	C11—H11	0.9300
C1—C2	1.427 (2)	C12—N1	1.320 (2)
C2—C3	1.351 (2)	C12—H12	0.9300
C2—H2	0.9300	C13—N3	1.4590 (18)
C3—C4	1.406 (2)	C13—C14	1.501 (2)
C3—H3	0.9300	C13—H13A	0.9700
C4—C5	1.410 (2)	C13—H13B	0.9700
C4—C9	1.425 (2)	C14—O1	1.4255 (19)
C5—N2	1.3539 (18)	C14—H14A	0.9700
C5—C6	1.447 (2)	C14—H14B	0.9700
C6—N1	1.3573 (19)	C15—N3	1.4574 (19)
C6—C7	1.415 (2)	C15—C16	1.509 (2)
C7—C10	1.400 (3)	C15—H15A	0.9700
C7—C8	1.427 (3)	C15—H15B	0.9700
C8—C9	1.340 (3)	C16—O2	1.415 (2)
C8—H8	0.9300	C16—H16A	0.9700
C9—H9	0.9300	C16—H16B	0.9700
C10—C11	1.361 (3)	O1—H4	0.8330
C10—H10	0.9300	O2—H5	0.9147
			
N2—C1—N3	116.99 (13)	N1—C12—H12	117.9
N2—C1—C2	121.66 (13)	C11—C12—H12	117.9
N3—C1—C2	121.35 (14)	N3—C13—C14	114.71 (12)
C3—C2—C1	119.07 (14)	N3—C13—H13A	108.6
C3—C2—H2	120.5	C14—C13—H13A	108.6
C1—C2—H2	120.5	N3—C13—H13B	108.6
C2—C3—C4	120.81 (14)	C14—C13—H13B	108.6
C2—C3—H3	119.6	H13A—C13—H13B	107.6
C4—C3—H3	119.6	O1—C14—C13	113.19 (13)
C3—C4—C5	116.60 (14)	O1—C14—H14A	108.9
C3—C4—C9	123.31 (15)	C13—C14—H14A	108.9
C5—C4—C9	120.08 (15)	O1—C14—H14B	108.9
N2—C5—C4	123.15 (14)	C13—C14—H14B	108.9
N2—C5—C6	118.39 (13)	H14A—C14—H14B	107.8
C4—C5—C6	118.45 (13)	N3—C15—C16	114.57 (13)
N1—C6—C7	121.82 (14)	N3—C15—H15A	108.6
N1—C6—C5	118.68 (13)	C16—C15—H15A	108.6
C7—C6—C5	119.50 (14)	N3—C15—H15B	108.6
C10—C7—C6	117.59 (16)	C16—C15—H15B	108.6
C10—C7—C8	122.76 (16)	H15A—C15—H15B	107.6
C6—C7—C8	119.65 (15)	O2—C16—C15	113.97 (13)
C9—C8—C7	120.76 (15)	O2—C16—H16A	108.8
C9—C8—H8	119.6	C15—C16—H16A	108.8
C7—C8—H8	119.6	O2—C16—H16B	108.8
C8—C9—C4	121.54 (16)	C15—C16—H16B	108.8
C8—C9—H9	119.2	H16A—C16—H16B	107.7
C4—C9—H9	119.2	C12—N1—C6	117.92 (14)
C11—C10—C7	119.93 (17)	C1—N2—C5	118.67 (12)
C11—C10—H10	120.0	C1—N3—C15	122.52 (12)
C7—C10—H10	120.0	C1—N3—C13	119.64 (12)
C10—C11—C12	118.52 (17)	C15—N3—C13	117.64 (11)
C10—C11—H11	120.7	C14—O1—H4	105.8
C12—C11—H11	120.7	C16—O2—H5	109.1
N1—C12—C11	124.22 (18)		
			
N2—C1—C2—C3	2.5 (2)	C6—C7—C10—C11	0.1 (3)
N3—C1—C2—C3	−177.39 (14)	C8—C7—C10—C11	179.74 (17)
C1—C2—C3—C4	−1.0 (2)	C7—C10—C11—C12	−0.3 (3)
C2—C3—C4—C5	−0.4 (2)	C10—C11—C12—N1	0.2 (3)
C2—C3—C4—C9	178.88 (15)	N3—C13—C14—O1	−56.55 (17)
C3—C4—C5—N2	0.5 (2)	N3—C15—C16—O2	−54.91 (18)
C9—C4—C5—N2	−178.84 (14)	C11—C12—N1—C6	0.2 (2)
C3—C4—C5—C6	179.25 (13)	C7—C6—N1—C12	−0.4 (2)
C9—C4—C5—C6	−0.1 (2)	C5—C6—N1—C12	179.63 (13)
N2—C5—C6—N1	−1.15 (19)	N3—C1—N2—C5	177.46 (12)
C4—C5—C6—N1	−179.99 (13)	C2—C1—N2—C5	−2.4 (2)
N2—C5—C6—C7	178.92 (13)	C4—C5—N2—C1	1.0 (2)
C4—C5—C6—C7	0.1 (2)	C6—C5—N2—C1	−177.83 (12)
N1—C6—C7—C10	0.3 (2)	N2—C1—N3—C15	177.43 (12)
C5—C6—C7—C10	−179.75 (14)	C2—C1—N3—C15	−2.7 (2)
N1—C6—C7—C8	−179.37 (14)	N2—C1—N3—C13	2.72 (19)
C5—C6—C7—C8	0.5 (2)	C2—C1—N3—C13	−177.39 (13)
C10—C7—C8—C9	179.05 (18)	C16—C15—N3—C1	−81.85 (17)
C6—C7—C8—C9	−1.3 (3)	C16—C15—N3—C13	92.96 (16)
C7—C8—C9—C4	1.3 (3)	C14—C13—N3—C1	−75.61 (17)
C3—C4—C9—C8	−179.92 (17)	C14—C13—N3—C15	109.43 (15)
C5—C4—C9—C8	−0.7 (3)		

### Hydrogen-bond geometry (Å, °)

**Table:**

*D*—H···*A*	*D*—H	H···*A*	*D*···*A*	*D*—H···*A*
O2—H5···O1^i^	0.91	1.91	2.8254 (17)	177
O1—H4···N1^ii^	0.83	2.08	2.8676 (18)	158

Symmetry codes: (i) −*x*−1, −*y*+2, −*z*; (ii) *x*−1, *y*, *z*.
